# Polarity during tissue repair, a multiscale problem

**DOI:** 10.1016/j.ceb.2019.07.015

**Published:** 2020-02

**Authors:** Alejandra Guzmán-Herrera, Yanlan Mao

**Affiliations:** 1 MRC Laboratory for Molecular Cell Biology, University College London, London WC1E 6BT, UK; 2 Institute for the Physics of Living Systems, University College London, London, UK; 3 College of Information and Control, Nanjing University of Information Science and Technology, Nanjing, Jiangsu 210044, People’s Republic of China

## Abstract

Tissue repair is essential for all organisms, as it protects the integrity and function of tissues and prevents infections and diseases. It takes place at multiple scales, from macroscopic to microscopic levels. Most mechanisms driving tissue repair rely on the correct polarisation of collective cell behaviours, such as migration and proliferation, and polarisation of cytoskeletal and junctional components. Furthermore, re-establishment and maintenance of cell polarity are fundamental for a tissue to be fully repaired and for withstanding mechanical stress during homeostasis and repair. Recent evidence highlights an important role for the interplay between cell polarity and tissue mechanics that are critical in tissue repair.

## Highlights

• Cell polarity is essential for repair across multiple scales.
• Polarised cell behaviours, cytoskeletal and junctional components drive repair.
• Polarity at the cellular level is crucial to restore and maintain tissue integrity.
• Cell polarity affects tissue mechanics and, in turn, how a tissue responds to injury.

## Graphical abstract

**Figure:**
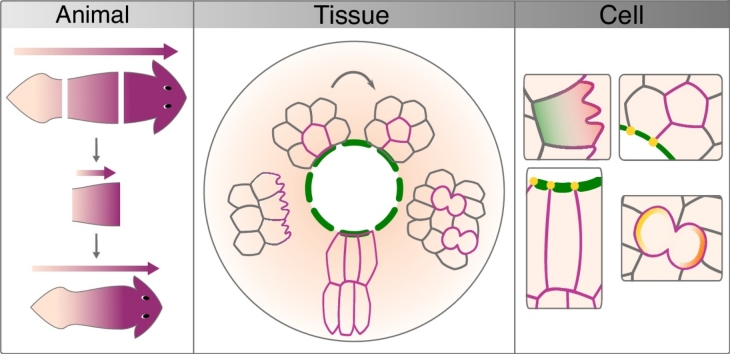

**Current Opinion in Cell Biology** 2020, **62**:31–36

This review comes from a themed issue on **Cell architecture**

Edited by **Sandrine Etienne-Manneville** and **Robert Arkowitz**

For a complete overview see the Issue and the Editorial

Available online 9th September 2019

**https://doi.org/10.1016/j.ceb.2019.07.015**

0955-0674/© 2019 The Authors. Published by Elsevier Ltd. This is an open access article under the CC BY license (http://creativecommons.org/licenses/by/4.0/).

## Introduction

Tissues consist of coordinated cells that form a particular shape with a specific structure and organisation. It is important that tissues are able to maintain their shape and structure throughout the lifespan of an organism, as well as to withstand and overcome damage. The ability of a tissue to restore injured structures in order to re-establish its integrity and continuity is known as tissue repair. It is required for preventing infections, via the entry of pathogens, as well as for maintaining tissue homeostasis and function [1,2]. Several mechanisms of tissue repair, in particular those underlying morphogenetic processes, are also observed in development [3, 4, 5, 6]. Additionally, when tissue repair is partially compromised or fails completely, it can lead to scarring, disease progression and even oncogenesis [7, 8, 9, 10]. Hence, understanding the processes involved in tissue repair and their regulation has significant implications for basic and translational research.

Tissue repair is required across all scales since injury events can range from macroscopic (missing or injured body parts) to microscopic levels (damaged cellular structures) [11,12]. The ability to repair at macroscopic scales, termed regeneration, is restricted to specific species, to certain body parts, or even periods in an individual’s lifetime [4,5,13, 14, 15, 16]. However, every organism has the capacity to repair at microscopic scales, as it is minimally required to maintain and restore tissue architecture upon any type of injury, whether mild or severe, caused by external or internal factors.

Cell polarity and its regulation have been shown to be an important feature of repair at all scales. From polarised cell behaviours to polarised distribution of cellular components, it is key for driving gap closure after injury and re-establishing tissue integrity and architecture. Furthermore, cell polarity is involved in preventing damage to maintain tissue homeostasis and, once damage has occurred, restricting its expansion to other areas or tissues. In this review we discuss some of the recent studies that highlight the important role of cell polarity during tissue repair across multiple scales.

## Polarity and repair at higher scales: whole body, limbs and large wounds

When extensive damage occurs, numerous cell types are depleted in large numbers, exposing underlying cells or tissues and compromising their function. Some examples of this include amputation events and large skin wounds that can be repaired to varying extents, from only closing the wound to regenerating the full structure [5,13,15]. If not resolved, this type of damage can temporally or permanently affect the fitness of the organism and its survival. At these higher macroscopic scales, polarisation of cells and of collective cell behaviours are essential to drive repair and successfully restore the damaged structures.

**Figure 1:**
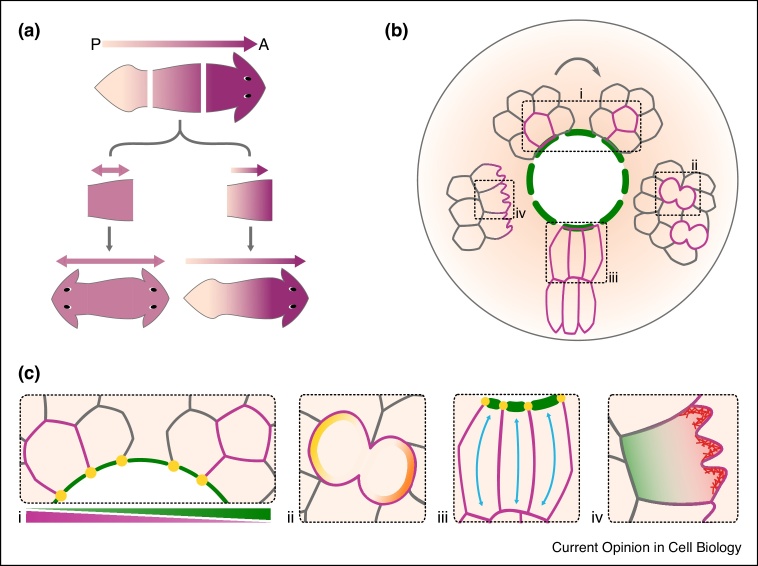
Repairing mechanisms across multiple scales. **(a)** AP body axis is established by bioelectrical signals that determine the correct position for head and tail regeneration [19,20]. **(b)** At a mesoscopic scale, many polarised cell behaviours take place to repair injured tissues, including (clockwise) wound edge cell intercalations [33^••^], oriented cell divisions [22], cell elongation [23,24], and cell migration [23,24]. **(c)** These polarised cell behaviours require subcellular polarisation of regulators and components of the cytoskeleton and the cell junctions, some of which are illustrated in this figure. (i) Higher wound edge intercalation rates (magenta) increase when myosin levels are lower, overcoming a weaker actomyosin purse-string [33^••^] (green). (ii) Cell division reorientation is driven by polarity components like Ft and Crb [25] (yellow and orange). (iii) Rock and JNK activity coordinates cell elongation towards the wound [23,24] (blue arrows). (i,iii) The actomyosin cable (green lines) forms at the wound edge and is intercellularly linked through stabilised AJs [2,38, 39, 40] (yellow dots). (iv) Polarisation of the cytoskeletal actin (red) and myosin (green) is required for cell migration [23,24].

In *Hydra*, supracellular actin fibres are polarised along the body axis. This actin organisation is inherited when a segment of the animal is amputated, determining the body axis of the regenerating animal. When discrepancies in the alignment of actin fibres emerge, multiple body axes are formed, resulting in an animal with multiple heads and feet [17^••^,^18^]. In planaria, the body axis is established by bioelectrical signals that trigger and regulate cell depolarisation and repolarisation immediately after injury [19,20]. Following body amputation, these signals establish anterior-posterior (AP) polarity of the remaining fragments, a crucial step for accurate head and tail regeneration. If AP polarity is lost, the amputated fragment grows two heads (one from each end) and no tail [20] (Figure 1a). In contrast, after hindlimb amputation in *Xenopus*, bioelectric signals promote cell depolarisation at the site of injury not only in the amputated limb but also in the undamaged contralateral limb [21^••^]. In the general case of wounded skin, the repairing process (known as re-epithelialisation) is driven by multiple polarised cell behaviours. It has been shown that a proliferating ring forms surrounding the damaged area but away from the wound edge. Within this ring, cell division is increased and oriented towards the wound in order to replenish the lost population of cells [22]. Simultaneously, to restore the initial stratified architecture of the skin, cells rearrange, proliferate, flatten, elongate and migrate in the direction of injury [22, 23, 24] (Figure 1b).

At the molecular level, several components that are relevant for establishing cell polarity have been implicated in the coordination and regulation of the polarised collective cell behaviours mentioned above (Figure 1c). For example, Rho kinase (Rock) and Jun N-terminal kinase (JNK) are needed to coordinate and direct various myosin-dependent cell rearrangements, such as cell flattening, radial cell intercalations, cell migration and cell elongation [23,24] (Figure 1c-iii,iv). Fat (Ft) and Crumbs (Crb) are known to reorient cell division and balance cell proliferation during regeneration (Figure 1c-ii). This reorientation is important to guide growth in the right direction, while balanced proliferation rates prevent the tissue from overgrowing [25]. As a result, the initial size and shape of the tissue can be restored. The actin cytoskeleton and its polarisation also play an essential role in repair since they influence multiple cell behaviours. Interestingly, it has been demonstrated that the circadian clock has a significant effect on the efficiency of actin-dependent processes, including cell migration and cell adhesion, by temporally regulating Rho, cofilin and other actin regulators. Consequently, both the circadian dynamics of actin and the time when injury takes place influence the efficacy of repair [26^•^].

## Polarity and repair at lower scales: epithelial tissues and cell architecture

In smaller wounds and in the early stages of the repair of larger ones [13,15], not only does the gap need to be completely closed but also, after this is accomplished, the original cell polarity and architecture need to be correctly restored. This is an essential step as it ensures the integrity and function of the tissue are recovered. The two main mechanisms responsible for the first step (sealing the gap) are (1) lamellipodia and filopodia formation, that allows opposing cells to come in contact with each other (Figure 1c-iv); and (2) the assembly of an actomyosin supracellular cable (also known as the purse-string) at the wound edge, which coordinates cell movements and reduces wound size through its contraction [2,27] (Figure 1c-i,iii). These mechanisms are not mutually exclusive and sometimes both are required to achieve efficient repair [2,27,28]. Importantly, the actomyosin cytoskeleton must be polarised for both types of structures to assemble and carry out their functions. Thus, for these two mechanisms to take place, it is essential to precisely regulate the dynamics of intercellular junctions and the polarisation and repolarisation of cytoskeletal and junctional components [2,29, 30, 31, 32,33^••^] (Figure 1c).

Two requirements for actomyosin accumulation and purse-string formation are Rock activity [24] and polarised endocytosis of molecular components, such as actin structures [34] and E-cadherin (E-cad) at the wound margin [29]. The latter has been shown to be triggered by calcium signalling [29] and reactive oxygen species (ROS) [32], a hallmark for damage [35, 36, 37]. Once it is assembled, the actomyosin purse-string needs to be maintained and stabilised throughout the process of wound closure. Adherens junctions (AJs) are responsible for linking the cable intercellularly [38, 39, 40] and it has been suggested that Echinoid (the nectin ortholog in *Drosophila*) is involved in stabilising those AJs and, therefore, the purse-string [2] (Figure 1c-i,iii).

## Cell polarity and tissue mechanics during repair

There is increasing evidence that polarisation of cytoskeletal and junctional proteins affects tension levels in the tissue, which in turn influences how cells behave in response to injury [18,30,31,33^••^]. It has been shown in the *Drosophila* embryo that increased tension and decreased myosin turnover, only at the wound edge, is also necessary for cable stabilisation [30]. Moreover, in the *Drosophila* imaginal wing disc, wound repair can be accelerated or hindered through the manipulation of myosin II (MyoII) activity and thus junctional tension [33^••^]. Even though inactivation of MyoII results in a weaker purse-string, this perturbation also reduces junctional tension, unjamming (fluidising) the tissue, promoting cell intercalations at the wound edge and accelerating wound closure. Conversely, hyperactivating MyoII increases tension, jamming the tissue and hindering repair [33^••^] (Figure 1c-i).

While the focus has mainly been on the role of AJs and their components during repair, other types of junctions have started to gain attention. Occluding junctions (OJs) are important for apicobasal polarity, as they separate the apical from the basolateral membrane compartment and seal the gaps between neighbouring epithelial cells. More recently, septate junctions (SJs), one type of OJs, have also been implicated in the regulation of epithelial tissue mechanics during repair. In the *Drosophila* embryo, perturbing the composition of SJs resulted in abnormal dynamics of actomyosin as well as AJ and SJ components at the wound edge, causing a defective wound closure [31].

In some cases, certain characteristics, such as cell polarity, packing and topology, need to be maintained and protected from morphogenetic forces while the tissue is being repaired. The actin cable can help protect cell packing and prevent scarring, as seen in gap closure in the *Drosophila* embryo [41], while thick actin belts can form to preserve cell topology and maintain the architecture of more complex tissues, as observed in mice cochlear epithelium [42]. Once the gap has been sealed, cell junctions need to form and mature to strengthen cell adhesion and recover the architecture of the tissue. Otherwise, tissue integrity and correct functioning remain compromised, leading to infections and pathology development [10,43^•^]. For successful maturation of junctions, tension-induced recruitment of myosin-1c to the lateral membrane is required to indirectly promote accumulation of adhesion proteins at the newly formed junction [44].

**Figure 2:**
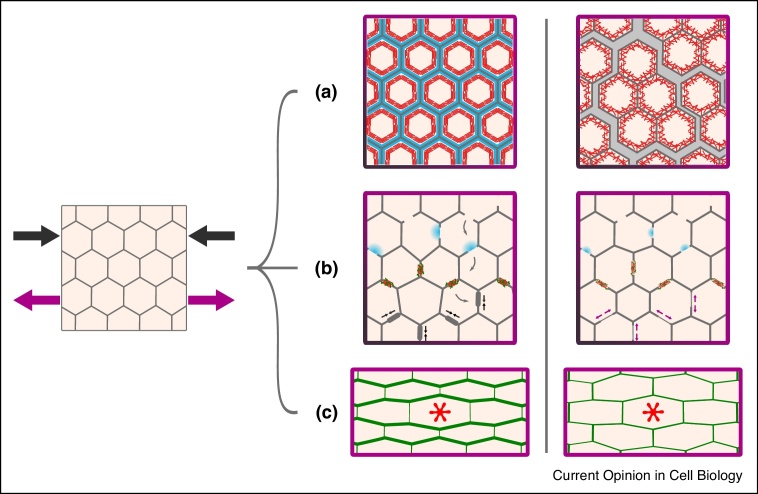
Protective and repairing mechanisms in response to mechanical stress rely on cytoskeletal and junctional polarisation. The constant presence of mechanical forces (black (compressing) and magenta (stretching) arrows) can compromise tissue integrity (right panels). **(a)** To cope with higher tension levels and prevent the tissue from fracturing (right panel), RhoA (blue) strengthens the AJs through actin assembly and alignment (red) [46^•^]. **(b)** When TJs are ruptured, flares of active Rho (blue) trigger actomyosin (red and green) accumulation to contract (black arrows) and reinforce the junctions (thicker grey segment). If this fails (right panel), the junctions expand (magenta arrows) and are not properly repaired [47^••^] (grey arrows indicate the sequence of events). **(c)** Upon stress, myosin cables are polarised (thicker green lines, left panel) to prevent drastic morphological changes and propagation of damage (right panel) [48^••^] (red star represents injury through laser ablation).

Even in the absence of external injury, normal cell behaviours, including cell movement, cell division and changes in cell shape and tension, can generate mechanical stress that accumulates in the cell junctions, making them prone to breakage [45,46^•^,47^••^]. Hence, proper maintenance of cell polarity is crucial for buffering mechanical stress, preventing cell damage from spreading and maintaining tissue integrity [43^•^,46^•^,47^••^,48^••^,^49^] (Figure 2). In response to increased tension in the tissue, RhoA is activated at the AJs, where it promotes actin assembly to enhance their tensile strength and prevent the epithelium from fracturing [46^•^] (Figure 2a). Mechanical stress on the cell edges can cause small but compromising ruptures at the tight junctions (TJs), a second type of OJs. These ruptures are immediately followed by a localised flare of Rho activation that triggers actomyosin accumulation, contracting the junction and concentrating TJ proteins to restore and reinforce it [47^••^] (Figure 2b). Accurate organisation of the actomyosin network in homeostatic conditions is also important to preserve the integrity of the tissue. It has been shown that a heterogeneous actomyosin distribution leads to hypercontractility at the tricellular junctions. This causes a basal displacement of the TJ belt along the lateral membrane, an expansion of the apical domain and results in a compromised barrier function [43^•^]. Furthermore, mechanical forces can cause undesirable changes in tissue morphology and abrupt propagation of small injuries. A protective mechanism recently described, consists of myosin cables polarised in the direction of stress that stiffen the tissue, limiting shape changes and containing possible damage while the mechanical forces are dissipated [48^••^] (Figure 2c). The recent studies summarised here highlight the relevance of the relationship between cell polarity and tissue mechanics, as well as its role in maintaining and repairing tissue architecture.

## Discussion and perspectives

Some organisms are able to cope with injury at higher scales, for which cell depolarisation and repolarisation are crucial to replenish the lost population and restore the initial architecture. In contrast, all organisms are able to repair at lower microscopic scales. This ability is fundamental for repairing any type of injury, mild or severe, and for maintaining tissue integrity during homeostasis. Most of the mechanisms implicated in microscopic repair rely on the correct polarisation of cytoskeletal and junctional components, an essential characteristic that is also relevant to tissue mechanics. Recent studies have begun to elucidate the relationship between cell polarity and tissue mechanics, as well as how both influence cell behaviours in response to injury and mechanical stress to repair and protect the tissue.

It is important to note that so far, most studies have examined cell polarity and repair in two dimensions; little work has focused on the role of apicobasal and basolateral polarity in tissue repair and how it might be affected along the process. Nonetheless, there has been an increasing interest in understanding the complex three-dimensional architecture of cells and tissues, how it is shaped, maintained and repaired. Moreover, the interplay between cell polarity and tissue mechanics, both in tissue homeostasis and repair, has started to gain more attention. With the help of novel experimental, computational and microscopy techniques, the coming years should further our understanding of these topics, in particular how they occur in three dimensions.

## Conflict of interest statement

Nothing declared.

## References and recommended reading

Papers of particular interest, published within the period of review, have been highlighted as:

• of special interest
•• of outstanding interest
